# Segment-targeted peroral endoscopic myotomy for recurrent diffuse esophageal spasm with giant diverticula

**DOI:** 10.1055/a-2792-9990

**Published:** 2026-03-11

**Authors:** Lin-da Li, Dong-Li He, Yun-Shi Zhong

**Affiliations:** 1 92323 Endoscopy Center, Zhongshan Hospital Fudan University, Shanghai, China; 2 Endoscopy Center, Xuhui Hospital, Affiliated Zhongshan Hospital, Fudan University, Shanghai, China

**Fig. 1:**
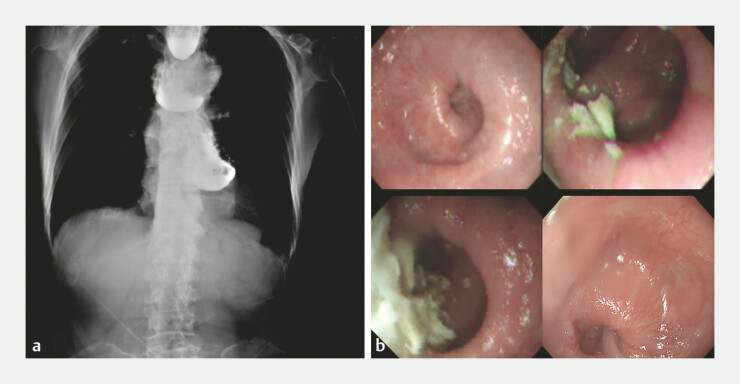
Imaging revealed multiple esophageal diverticula and hypercontractile segments. Barium study showing multiple diverticula. An endoscopic view revealing diverticula and hypercontractile rings.

A 73-year-old male patient presented with recurrent dysphagia 6 years after prior peroral endoscopic myotomy (POEM) for type III achalasia. The recurrent symptoms were mainly attributed to diffuse esophageal spasm coexisting with residual achalasia. Imaging revealed multiple thoracic esophageal diverticula associated with hypercontractile segments ( Fig. 1 ).

**Fig. 2:**
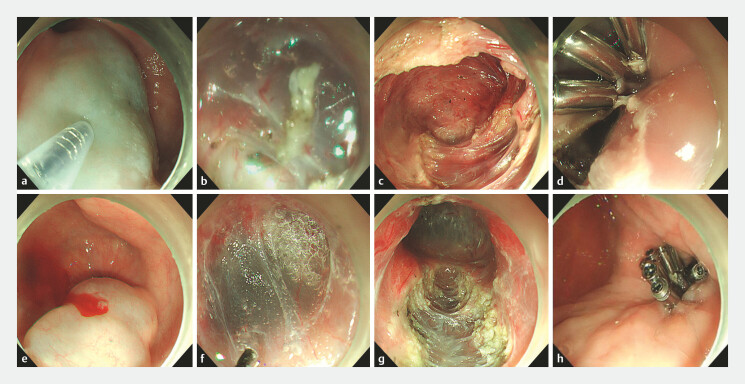
Sequential steps of two-segment myotomy and closure with clips. **a** Establishment of the posterior tunnel entry to avoid interference from the previous POEM scar, and submucosal injection revealed poor mucosal lifting. **b** Subsequent incision demonstrated a whitish, irregular, muscle-like submucosal appearance. **c** A full-thickness myotomy of both circular and longitudinal muscle layers extending 5 cm. **d** Mucosal incisions were closed with titanium clips. **e** Establishment of the tunnel entry at 23 cm from EGJ and submucosal injection revealed good mucosal lifting. **f** Subsequent incision demonstrated a transparent blue layer. **g** A full-thickness myotomy of both circular and longitudinal muscle layers extending 5 cm. **h** Mucosal incisions were closed with titanium clips. POEM, peroral endoscopic myotomy.

A modified POEM was employed to carry out two selective full-thickness myotomies through short submucosal tunnels. Each myotomy was precisely targeted at the hypercontractile rings related to the esophageal diverticula. Through a distal tunnel (with an entry 13 cm from the EGJ and extending 7 cm), the hypercontractile ring at the distal diverticulum was dissected ( Fig. 2 **a–d** ; Video 1 ). Subsequently, a proximal tunnel (entry at 23 cm from the EGJ, extending 7 cm) was created to perform myotomy on the corresponding hypercontractile segment ( Fig. 2 **e–h** ). All myotomies were performed as full-thickness procedures using a hybrid knife, and the mucosal entry sites were secured with titanium clips. The patient had an uneventful recovery and remained symptom-free during the 10-month follow-up.

The first POEM at 13 cm created a tunnel to address severe fibrosis, complex anatomy, and a contractile ring, enabling diverticulum treatment. POEM, peroral endoscopic myotomy.Video 1

We have developed a novel segmental repetitive puncture endoscopic treatment technique, which can effectively establish multiple tunnels for sigmoid-type esophagus 1 and severe submucosal fibrosis 2 , and can also specifically separate the contractile ring serving as the diaphragm of the diverticulum 3 , thereby offering an individualized and effective solution for technically challenging recurrent jackhammer esophagus.

Endoscopy_UCTN_Code_TTT_1AO_2AP
